# Dynamic auxin transport patterns preceding vein formation revealed by live-imaging of Arabidopsis leaf primordia

**DOI:** 10.3389/fpls.2014.00235

**Published:** 2014-06-11

**Authors:** Danielle Marcos, Thomas Berleth

**Affiliations:** Department of Cell and Systems Biology, University of TorontoToronto, ON, Canada

**Keywords:** Arabidopsis, auxin transport, leaf vein formation, live confocal imaging, pattern formation, PIN1, systems biology, plant vascular development

## Abstract

Self-regulatory patterning mechanisms capable of generating biologically meaningful, yet unpredictable cellular patterns offer unique opportunities for obtaining mathematical descriptions of underlying patterning systems properties. The networks of higher-order veins in leaf primordia constitute such a self-regulatory system. During the formation of higher-order veins, vascular precursors are selected from a homogenous field of subepidermal cells in unpredictable positions to eventually connect in complex cellular networks. Auxin transport routes have been implicated in this selection process, but understanding of their role in vascular patterning has been limited by our inability to monitor early auxin transport dynamics *in vivo*. Here we describe a live-imaging system in emerging *Arabidopsis thaliana* leaves that uses a *PIN1:GFP* reporter to visualize auxin transport routes and an *Athb8:YFP* reporter as a marker for vascular commitment. Live-imaging revealed common features initiating the formation of all higher-order veins. The formation of broad *PIN1* expression domains is followed by their restriction, leading to sustained, elevated *PIN1* expression in incipient procambial cells files, which then express *Athb8*. Higher-order *PIN1* expression domains (hPEDs) are initiated as freely ending domains that extend toward each other and sometimes fuse with them, creating connected domains. During the restriction and specification phase, cells in wider hPEDs are partitioned into vascular and non-vascular fates: Central cells acquire a coordinated cell axis and express elevated *PIN1* levels as well as the pre-procambial marker *Athb8*, while edge cells downregulate *PIN1* and remain isodiametric. The dynamic nature of the early selection process is underscored by the instability of early hPEDs, which can result in dramatic changes in vascular network architecture prior to *Athb8* expression, which is correlated with the promotion onto vascular cell fate.

## Introduction

The vascular system of plants is a network of strands composed of xylem and phloem that transport water and photoassimilates, respectively (Esau, 1965). In leaf primordia, xylem and phloem both differentiate from procambial precursors: long, narrow, cytoplasm-dense cells, (Esau, 1965) arranged in continuous strands (Esau, 1943). In leaves, procambial strands arise from continuous files of isodiametric “preprocambial” cells, which are selected from the anatomically homogeneous subepidermal tissue of the leaf primordium (Foster, 1952; Pray, 1955; Scarpella et al., 2004). Procambial cells subsequently acquire their characteristic narrow shape through coordinated, oriented cell divisions parallel to the axis of the strand (Foster, 1952; Esau, 1965).

The mechanism by which preprocambial cells are selected to the vascular cell fate is not known, but among potential network-forming mechanisms that have been proposed and mathematically investigated (reviewed in Berleth et al., 2007), a link to preferred routes of auxin transport has long gained experimental support (Sachs, 1969, 1981, 1989; Sauer et al., 2006). Auxin is unique among plant hormones as it is actively transported from cell to cell in a polar fashion (Blakeslee et al., 2005). Experimental visualization of auxin transport routes in early organ primordia and their potential role in vascular patterning requires markers of auxin transport and vascular cell selection. Expression levels and subcellular localization of auxin transport facilitators of the *PIN* family control the magnitude (Petrasek et al., 2006) and direction (Wisniewska et al., 2006) of auxin flow, respectively (reviewed in Zazimalova et al., 2007). *Athb8* encodes a member of homeodomain-leucin zipper (HD-ZIP) III family of putative homeobox transcription factors (Ruberti et al., 1991; Schena and Davis, 1992; Sessa et al., 1998; Baima et al., 2001). The gene has been functionally implicated in early vascular development and is auxin-inducible as a direct target of the Auxin Response Factor (ARF) MONOPTEROS (MP, ARF5) (Hardtke and Berleth, 1998; Donner et al., 2009). Importantly, expression of *Athb8* has been shown to be the most reliable marker of pre-procambial cell fate (Scarpella et al., 2004).

In *Arabidopsis thaliana*, veins are classified according to a hierarchical order, starting from a midvein (first order), followed by reproducibly positioned, looped side-branches (second order) and then by variable branches of 3, 4, and 5th order, the latter collectively referred to as the higher-order vein network (Nelson and Dengler, 1997). In addition to vein order, veins can be further categorized by their degree of connection to other veins. Freely-ending veins are connected to another vein only at one end, while connecting veins are attached to another vein at both ends. Visualizing gene expression domains in staged leaf primordia, the ontogeny especially of veins of the first two orders and their link to auxin transport routes has been reconstructed at high precision (Scarpella et al., 2006). However, studies on staged primordia cannot establish general developmental principles for the majority of veins, as they cannot visualize the ontogeny of higher-order veins. Those arise in unpredictable positions and thus have to be observed continuously through live imaging in individual primordia as they form.

In this study, existing plant live imaging protocols (Boisnard-Lorig et al., 2001; Grandjean et al., 2004; Reddy et al., 2004; Heisler et al., 2005; Campilho et al., 2006; Xu et al., 2006; Sawchuk et al., 2007), were modified to allow for monitoring gene expression domains of internal organs at high subcellular resolution and without interference with the normal course of development. The technique was tested for its non-obstructive influence on normal vein development (Marcos and Berleth, unpublished data) and used to monitor the expression and subcellular localization of the auxin transport facilitator PIN1 relative to the auxin response reporter *DR5*, the pre-procambial marker *Athb8* and histological markers of cell identity in a series of non-overlapping samples. In the emerging picture, the selection of vascular precursors is initiated by highly dynamic and partly reversible prepatterns of apparent auxin transport routes, followed by the gradual restriction of those routes and the specification of procambial cell identity at sites of sustained auxin transport within narrow domains of *PIN1* expression.

## Materials and methods

### Plant material

The origin of *PIN1:GFP* and *DR5rev::GFP* has previously been described (Benkova et al., 2003; Friml et al., 2003). The homozygous *Athb8::YFPer* line was a kind gift from Prof. Ben Scheres. *Athb8::YFPer PIN1:GFP* double marker lines were generated by fertilizing emasculated flowers of *PIN1:GFP* homozygous plants with pollen from homozygous *Athb8::YFPer* plants, and selecting progeny homozygous for both markers.

### Culture system for live imaging

Seeds were sterilized as described (Scarpella et al., 2004), and sown on 100 μL growth medium in a single depression slide. The slide was placed in a culture plate containing 25 mL 0.8% (w/v) agar. Plates were sealed with micropore tape (3M, VWR Intl., Mississauga, ON, CAN), to prevent desiccation. Plates were stratified in the dark at 4°C for 5 days, and then incubated at 25°C under continuous fluorescent light (100 μE m^−2^ s^−1^). Seedling leaf primordia were visualized beginning at 3 DAG. “Days after germination” (Christensen et al., 2000) are defined as days after exposure of imbibed seeds to light. For confocal imaging, the depression slide carrying the seedling was removed from the culture plate. The seedling was mounted on the depression slide under a coverslip, using sterile double-distilled water as the mounting medium. After visualization of the first leaf primordium, the coverslip was removed and the depression slide returned to the sealed culture plate. Leaves were imaged at intervals of 8–12 h, for a total maximum duration of 72 h. After the imaging period, selected seedlings were transferred to Promix BX growing medium (Premiere Horticulture, Ref Hill, PA, USA) in 7 × 7 × 8 cm pots at the approximate density of 0.1 seedling per cm^2^ and grown under fluorescent light (100 μE m^−2^ s^−1^) for a 16-h light cycle at 22°C, followed by an 8-h dark cycle, 18°C.

### Microtechniques and microscopy

*PIN1:GFP* and *PIN1:GFP DR5rev::VENUS:N7* samples were observed with a Zeiss Axiovert 100 M confocal microscope equipped with a Zeiss LSM510 laser module confocal unit (Carl Zeiss, Oberkochen, Germany). GFP and VENUS were visualized with the 488 nm line of an Argon laser at 25% of output and 4–10% transmission, and with either a 505–530 or a 500–550 nm band-pass filter. *PIN1:GFP Athb8::YFPer* samples were observed with a Zeiss LSM510 META laser module laser confocal microscope (Carl Zeiss, Oberkochen, Germany). GFP was visualized with the 488 nm line of an Argon laser at 23% transmission, with a 505–530 band-pass filter; YFP was visualized using the 514 nm laser line of the same Argon laser at 17% transmission, with a 535–590 band-pass filter. Signal-to-noise ratio was increased during image acquisition by 2-frame averaging (Russ, 1995), and post-acquisition by neighborhood averaging with a 9 × 9 Gaussian kernel with standard deviation radius of 1 pixel (Russ, 1995).

For visualization of the final xylem pattern, first leaves were fixed in ethanol:acetic acid (3:1, v/v) for 24 h at room temperature. Samples were stored in 70% (v/v) ethanol at 4°C. For microscopy, rehydrated samples were dissected under water, mounted abaxial side up in chloral hydrate:glycerol:water (8:3:1, w/v/v) and viewed under dark-field illumination (Leica MZFLIII microscope, Leica Microsystems GmbH, Wetzlar, Germany). Images were acquired with a Canon EOS D60 digital camera (Canon Inc., Tokyo, Japan).

### Image analysis and processing

*PIN1* intensity data was obtained from samples expressing only the *PIN1:GFP* transgene by measuring the GFP signal intensity values along the proximal-distal axis of each freely-ending hPED using the Profile tool of the imaging software for the Zeiss LSM510 confocal microscope (Carl Zeiss, Oberkochen, Germany). Only differences in signal intensity that were greater than 20 relative units were counted as different.

Cell length data was obtained by measuring the length of cells along the long axis of each hPED, using Zeiss LSM Image Browser software (Carl Zeiss, Oberkochen, Germany). Only differences in cells of lengths that were greater than 1 μm were counted as different. The hPED strand was defined as the cells between (a) and (b), where (a) is the point of connection of the hPED to the lower order vein, and (b) can be either the: (i) point of connection to a second lower order PED in the case of a connected hPED, or (ii) the branch point from which a second hPED emerged or (iii) the free end of the hPED, whichever occurred first. It was noted that the cell linking the hPED to the connecting lower order PED remained shorter than its neighboring cell (by more than 1 μm) in 72% of cases (58/81 hPEDs). This suggests that this cell is under special developmental constraints due to its unique position as a linker and as such its vascular differentiation might be somewhat uncoupled from that of the rest of the strand. This is consistent with observations that cells in this linker position also differentiate into xylem later than the rest of the strand (E. Scarpella, pers. comm). Due to these observations, the length of the linker cell was omitted when determining the cell length gradient along the proximal-distal axis of the hPEDs.

All images for figures were assembled using Adobe Photoshop 7.0 (Adobe Systems, Mountain View, CA, USA).

## Results

### *PIN1* expression dynamics during arabidopsis higher-order vein pattering

**Figure 1:**
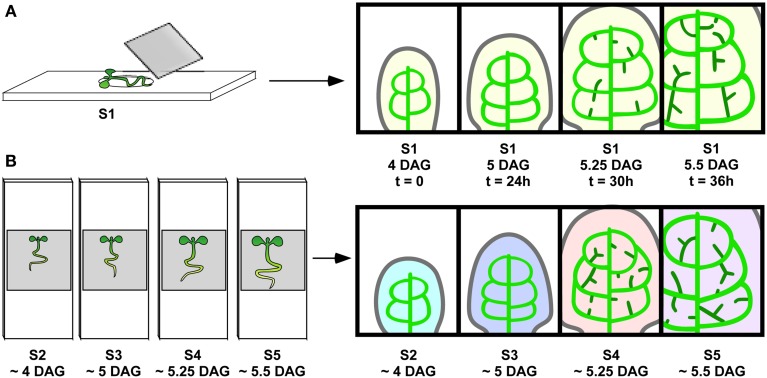
**Live imaging vs. reconstruction of vein ontogeny from staged samples. (A)** Live imaging. Sample S1 (left) growing in growth medium on depression slide and put under cover slip for visualization at intervals tested for not interfering with vein development to produce a series of images (right). Since the same veins are visualized repeatedly over time, one can follow the development of the randomly positioned hPEDs. **(B)** Reconstruction from staged samples. Synchronously grown samples, S2, S3, S4, and S5, are mounted and visualized, resulting in one image per sample (indicated by different colors). This approach is appropriate for vein classes (one, midvein) and (two, second order vein loops), where a reproducible vein pattern allows for the unambiguous identification of each vein across samples.

The unique properties of the PIN1:GFP fusion protein for visualizing presumptive auxin-transport routes specifically in leaf primordia has previously been demonstrated (Scarpella et al., 2006) and has been further supported by genetic studies (Sawchuk et al., 2013). In order to describe the dynamics of the predicted auxin transport routes, as inferred from *PIN1* expression domains, preceding the emergence of higher-order veins, we developed a live imaging system to visualize *PIN1* expression domains (PEDs) in individual, growing Arabidopsis first leaf primordia from 3 to 5 days after germination (DAG; Figure 1). Leaves imaged at 8–24 h intervals attained a significantly smaller final size than controls: Mean final leaf area equaled 9.23 ± 6.79 mm^2^ for imaged leaves (*n* = 73) vs. 15.67 ± 9.10 mm^2^ for non-imaged controls (*n* = 58; *P* = 1.92 × 10^−5^). As this raised the possibility that altered leaf growth dynamics could change PED dynamics, we compared the developmental outcome of conventional and live-imaging visualization of second-order PED formation. We examined its emergence under live imaging conditions and found that in 20 out of 22 live-imaged primordia, the ontogeny of the second loop PED was found unchanged relative to that observed in singly imaged samples (Scarpella et al., 2006). As an additional control, live-imaged primordia were cultured post-visualization, allowing the comparison of veins in the PED pattern with those in the final xylem pattern. The midvein, second order loops and most higher-order veins observed in the *PIN1* expression stages during the live imaging interval could be traced in the final xylem pattern, and the xylem pattern of these veins appeared normal (Figures 1A,B; See also Figures 4F–J). Since neither PED patterns nor subsequent vascular differentiation was affected in the vast majority of live imaging samples, we used this visualization protocol to characterize PED dynamics during the formation of higher-order veins.

**Figure 2:**
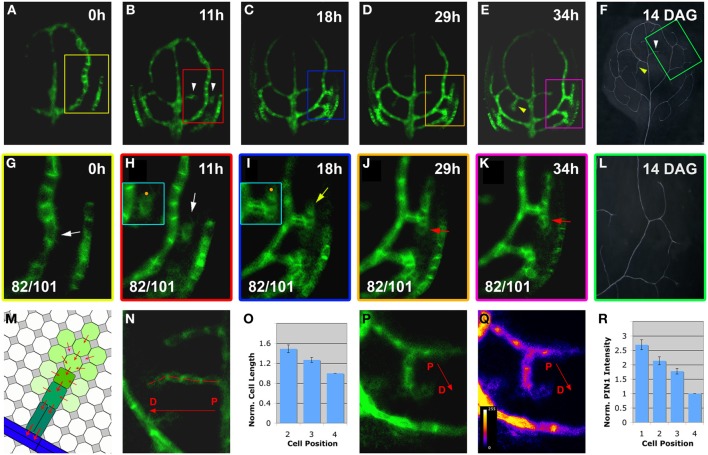
**Live-imaging of hPED initiation and extension**. Top right, time after first imaging time point (*t* = 0; **A–E**,**G–K**) or DAG **(F,L)**. Bottom left, reproducibility quotient (Table 2). Scale bars: **(A–E)** 20 μm; **(F,L)** 1 mm; **(G–K,N–Q)** 10 μm. **(A–F)** *PIN1:GFP* expression in a first leaf from 3 to 4.5 DAG. Note the appearance of freely-ending hPEDs in **(B)** (white arrowheads) and in **(E)** (yellow arrowhead) Matching higher order veins in leaf at 14 DAG marked by arrowheads in **(F)** and boxed area enlarged in **(L)**. **(G–L)** Detail of *PIN1:GFP* expression and xylem pattern in the boxed areas shown in **(A–E,F)**, respectively. Note weak *PIN1* expression in cells adjacent to the first loop (white arrow in **G**) initiating higher-order vein. Upregulated *PIN1* in the neighbours of one of these cells (white arrow in **H**). PIN1 localization is directed toward the center of the hPED (“central”) and/or directed toward the lower order loop (“basal”; turquoise-boxed inset in **H**). The hPED is extended through the upregulation of *PIN1* in additional cells at the free end of the hPED **(J,K)**. The first recruited cells in the “proximal” part of the hPED (nearest to the connecting lower order vein) further upregulate *PIN1* (yellow arrow in **I**), and elongate and/or divide along the predicted axis of auxin flow, becoming procambium (red arrowheads in **J,K**). The final vein pattern of the same leaf area is shown in **(L)**. **(M–R)** Gradient of cell length **(N)** and *PIN1* expression (**P**, intensity monitored in **Q**) along the proximal-distal axis of the hPED (labeled with a red arrow in **N,P,Q**). Schematic gradient in **M** green shading reflects *PIN1* expression levels, red arrowheads PIN1 polarity. **(O)** graph of average normalized cell length of the 2, 3, and 4th cells of the hPED (*n* = 42). Cell length values were normalized against the cell length value of the 4th cell for each hPED. **(R)** graph of average normalized *PIN1* intensity of the first four cells of the hPED (*n* = 16). *PIN1* intensity values were normalized against the *PIN1* intensity value of the 4th cell for each hPED. Error bars in **(O,R)** show the standard error. (See Methods for details).

**Figure 3:**
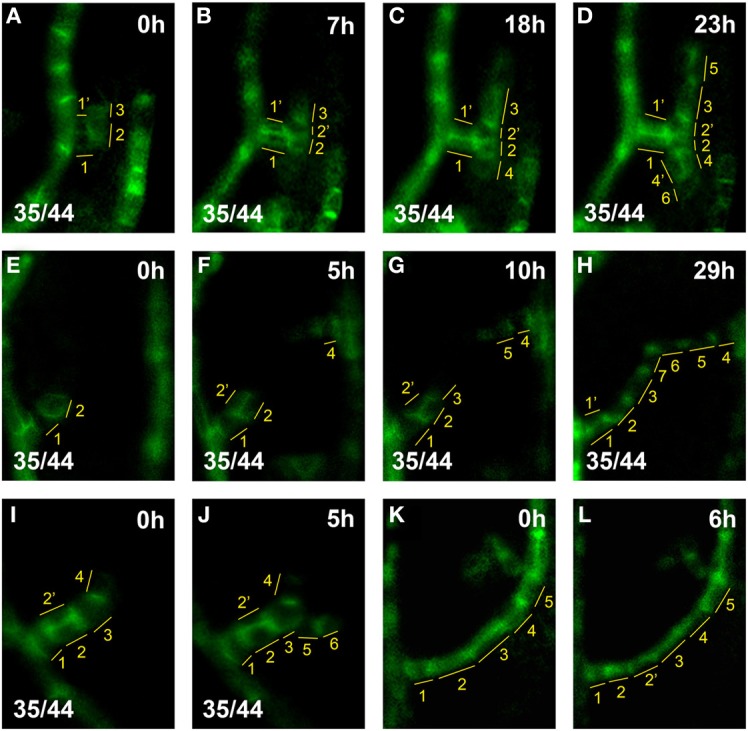
**hPED extension by terminal addition vs. intercalation**. Top right, time after first imaging time point. Bottom left, reproducibility quotient (Table 2). Scale bars: **(A–L)** 10 μm. **(A–D)** hPED extension via terminal addition: At 0 h, a freely-ending hPED of four cells branching from the first loop (**A**, cells numbered in yellow). By 18 h, Cell 4, next to Cell 2, upregulated *PIN1*. By 23 h, Cells 5 and 6 were also added to the hPED, next to Cells 3 and 4, respectively. During the live imaging period, Cells 2′ and 4′ were also added, via the divisions of Cell 2 (at 7 h) and Cell 4 (at 23 h), respectively. However, these cell divisions did not increase the length, but rather the width, of the PED. **(E–H)** PED extension and fusion via terminal addition: At 0 h, a freely-ending hPED branching from the first loop (**E**, cells numbered in yellow). By 5 h, Cell 4, has upregulated *PIN1* generating a new freely-ending hPED branching from midvein. By 10 h, Cell 3 has been added next to Cell 2 in the lower hPED, and Cell 5 added next to Cell 4 in the upper hPED. By 29 h, Cells 6 and 7 have also been added, resulting in the fusion of the two hPEDs. Note that Cell 2′ was also added to the hPED at 5 h by the division of Cell 2, widening the hPED in that area to 2-cells wide. By 29 h, Cell 2′ no longer expresses *PIN1*, and the hPED has again become 1-cell wide at that spot. (**I–J)** Extension of a new hPED branch via terminal addition: At 0 h, freely-ending hPED composed branching from the first loop (**I**, cells numbered in yellow). By 5 h, Cells 5, 6 upregulated *PIN1* an hPED branch. **(K,L)** Extension of loop PED by cell intercalation: At 0 h, the lower part of loop PED five cells long (**K**, cells numbered in yellow). By 6 h, Cell 2 division generates Cell 2′. Note that intercalation is not observed in hPEDs.

**Figure 4:**
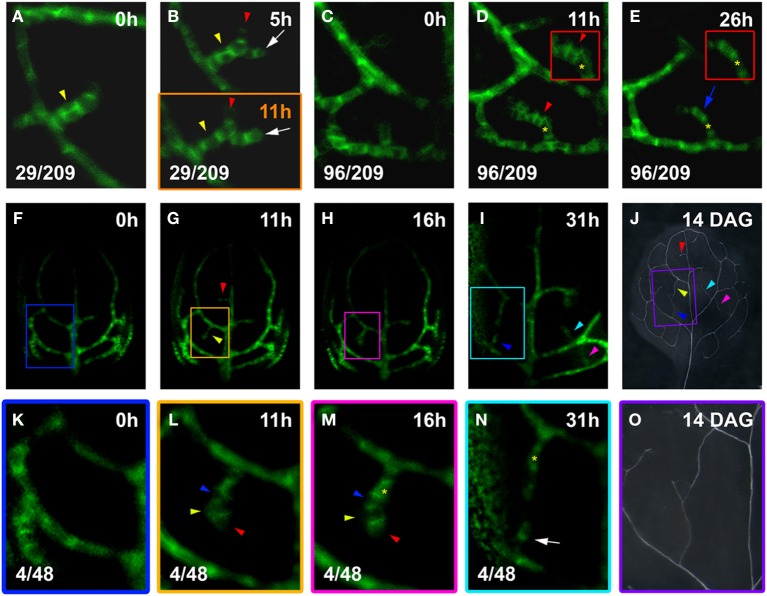
**hPED branching and narrowing**. Top left, time after first imaging (*t* = 0; **A–I**,**K–N**) or at DAG **(J,O)**. Bottom left, reproducibility quotient (Table 2). Scale bars: (**A–E**,**K–N**) 10 μm; **(F–I)** 20 μm; (**J,O)** 1 mm. **(A,B)** Freely-ending four-cell hPED **(A)**, by 5 h extended by one cell (red arrowhead, **B**) and associated with side branch (white arrow). By 11 h (**B**, lower panel), cells of the new side branch (white arrow) elongated with increased *PIN1* expression. Yellow arrowheads in **(A,B)** indicate where hPED is originally two cells wide (**A,B** upper panel). By 11 h (**B**, lower panel), *PIN1* expression has been lost by the upper cell (yellow arrowhead), resulting in a hPED that is uniformly 1-cell wide. **(C–E)** At 0 h **(C)**, a freely-ending hPED extends from the third loop PED. By 11 h, *PIN1* upregulated in a group of cells (red arrowhead). Upper cells of this new domain (see red-boxed inset in **D**), display PIN1 polarity toward hPED center (red arrowhead). Lost *PIN1* expression in these same cells (blue arrow, **E**), while cells beneath maintained expression and elongated (red-boxed inset in **E**). Same-cell mark (yellow asterisk, **D,E**), as a positional reference. **(F–J)** *PIN1:GFP* expression and final vein pattern **(J)**, illustrating appearance of two and three freely-ending hPEDs at 11 h and 31 h, respectively (arrowheads in **G,I**, respectively) and corresponding veins (arrowheads in **J**). **(K–O)** Detail of *PIN1:GFP* expression in the boxed areas in **(F–I)**. Detail of the xylem pattern in boxed area in **(J)** is shown in **(O)**. No hPEDs are present in the area enclosed by the second loop at 0 h **(K)**. By 11 h **(L)**, a freely-ending hPED has formed that extends from the first loop and is four cells long. By 16 h **(M)**, the cells on the right side of the hPED have upregulated *PIN1* and localize it strongly to the basal cell membrane (red arrowheads in **L,M**). In contrast, the cells on the left side of the PED have decreased *PIN1* expression (yellow arrowheads in **L,M**) or localize PIN1 toward the center of the hPED (blue arrowheads in **L,M**). By 31 h **(N)**, the cells on the left side of the hPED have lost *PIN1* expression, resulting in the narrowing of the hPED (yellow asterisk marks same cell in **M,N**). Note the new freely-ending hPED from second loop PED at 31 h (white arrow in **N**). The upper and lower freely ending hPED in **(N)** fused to form a connected higher order vein in the final xylem pattern **(O)**.

As our study focused on the formation of the higher-order vein network, we introduced hPED as another term specifically referring to PEDs that are associated with the formation of veins of the 3rd and higher orders. When following a total of close to 100 hPEDs at regular intervals in living primordia an invariable pattern of emergence was visible: They were initiated as freely ending domains attached to pre-existing PEDs, and they extended into the growing leaf blade over time (Figures 2A–E; See also Figures 4F–I, 6A–D). During the early stages of hPED formation that we examined, extension predominantly arose from the upregulation of *PIN1* in naïve cells at the free end of the hPED (“terminal addition,” Figure 1). In most hPEDs, terminal addition could be distinguished from intercalation, as individual cell(s) could usually be identified across successive visualizations on the basis of their shape and arrangement (Figures 3A–J vs. 3K–L). Out of 44 extending, freely-ending hPEDs observed, 35 (79.5%) were extended by terminal addition, while in 9 cases (20.5%) the mode of extension was unclear. Thus, intercalation cannot be a major contributor to PED extension in 3–5 days old leaf primordia and, quite possibly, may not occur at all (See Discussion).

At their first appearance, hPEDs were composed of polygonal, isodiametric cells that weakly express *PIN1* (White arrows in Figures 2G,H, enlarged in turquoise inset in H). In all observed freely ending hPEDs, PIN1 subcellular localization indicated auxin flow from the hPED to the connecting low order PED (Figures 2G–K, enlarged in inset in I; See also Figures 4K–M, 6K–M). Subsequently, these cells began to elongate (Figure 2I, turquoise inset) and to divide in parallel to the predicted axis of auxin flow, thus going from polygonal to more square or rectangular in shape and thereby became anatomically recognizable as procambium. In these earliest procambial cells, *PIN1* was upregulated and strongly localized to the basal cell membrane (Figures 2H vs. 2I, turquoise insets; The same cell is indicated by a orange dot in both insets). (“Basal” is here defined as oriented toward the connecting lower order PED). This correspondence of *PIN1* upregulation with cell elongation during the preprocambium-procambium transition was observed in 71 out of 91 hPEDs.

As the cells of the hPED underwent this transition, *PIN1* was repeatedly upregulated in new isodiametric cells located at the free end of the strand, leading to the extension of the hPED (Figures 2H–K; See also Figures 3A–J). Thus, not all cells within a given extending hPED were at the same developmental stage: The cells recruited earliest, near the proximal point of connection to the lower order PED, were frequently more elongated and express *PIN1* more strongly than newly recruited cells at the distal free end of the hPED (Figures 2H–K,N,P). Thirty-seven out of 56 freely-ending hPEDs (66%) showed a completely uniform proximal to distal (P-D) *PIN1* intensity gradient at first appearance, such that *PIN1* intensity increased progressively in successive cells going from the older cells at the proximal base of the hPED to the most recently added cells at the distal free end (Figures 2P–R). In 10 hPEDs (18%), the *PIN1* signal intensity gradient was not uniform (i.e., one or more cells did not conform to the overall *PIN1* expression gradient, which was in most cases P-D) and five hPEDs (9%) showed no *PIN1* expression gradient (i.e., all cells of the hPED were of equal *PIN1* signal intensity). A reverse distal to proximal (D-P) *PIN1* expression gradient, wherein the most recently added cells at the free end of the hPED had stronger *PIN1* signal intensity than older cells at the base of the hPED, was never observed. Similarly, fifty out of eighty-one freely-ending hPEDs (62%) showed a uniform proximal-distal cell length gradient, such that cell length increased progressively going from the oldest, proximal cells near the hPED base to the most recently added cells at the distal end of the hPED (Figures 2N,O, schematic in M, compare Figure 8). In 15 hPEDs (18%), the cell gradient was not uniform (i.e., one or more cells was of a length that did not conform to the local hPED length gradient, which was in most cases an overall P-D), and eleven hPEDs (14%) showed no cell length gradient along their proximal-distal axis (i.e., all cells of the hPED were of equal length). Five hPEDs (6%) exhibited a reverse distal to proximal (D-P) length gradient, wherein the most recently added cells at the end of the hPED were longer than older cells at the base of the hPED. These data suggest that cell recruitment into an auxin transport route is followed by a progressive phenotypic transformation, such that a cell's shape and auxin transport capacity depends upon how long this cell has been part of the auxin transport route.

### hPED ontogenies in different leaf areas

hPEDs were not formed exclusively as narrow domains (1–2 cells wide) that extended in only one direction. Instead, a hPED could be initially two or more cells wide and a new hPED could form on an existing hPED while the original hPED was still and freely ending (Figure 4). Of the hPEDs formed in the first leaf 14% (29/209) were adjoined by a still higher order hPED while still freely ending. These branched, freely-ending hPEDs displayed very different levels of *PIN1* expression and different rates of strand elongation (Figures 4A,B).

Additionally, 46% of hPEDs observed (96/209) arose as wider PEDs (>2-cell wide) that narrowed over time, to a final width of only 1- to 2-cells (Figures 4C–E). Narrowing of hPEDs was accompanied by central (toward the center of the hPED) or centrobasal (between central and basal) PIN1 localization in cells that were not clearly in the center of the hPED (Figure 4D red arrowhead and inset). This was contrasted by predominantly basal PIN1 localization in cells in the central part of the hPED or all cells in single-cell wide hPEDs (Figures 4D vs. 4E, insets). Using live imaging at short time intervals, we followed in detail how this narrowing process occurs: Over time, edge cells increasingly localize PIN1 toward neighboring central cells that concomitantly upregulate *PIN1* and localize it preferentially to the basal membrane (Figures 4L vs. 4M). Expression of *PIN1* persists in the central cells, which further elongate and eventually become a procambial strand (Figures 4M–O). Cells at the edge of the hPED eventually lose *PIN1* expression (Figures 4M vs. 4N). hPEDs thus have a tendency toward an ultimate width of only one cell. If, in a 1-cell wide hPED, a cell division parallel to the axis of the strand generates a spot 2-cells wide (Figures 4A,B; See also Figures 6K–N), centrobasal PIN1 localization in the two daughter cells is followed by decreased *PIN1* expression in one of them (Figures 4A,B; See also Figures 6K–N), indicating a persistent mechanism of hPED self-restriction to minimal widths. This observation is consistent with earlier reports that vascular precursors in the developing leaf occasionally divide to produce one vascular daughter cell and one non-vascular daughter cell (Pray, 1955). Taken together, these results indicate that the elaboration of the leaf vascular network proceeds from relatively widespread *PIN1* expression in many connected, wide hPEDs to general hPED narrowing, with procambium formation occurring in the cell files with sustained *PIN1* expression.

**Figure 5:**
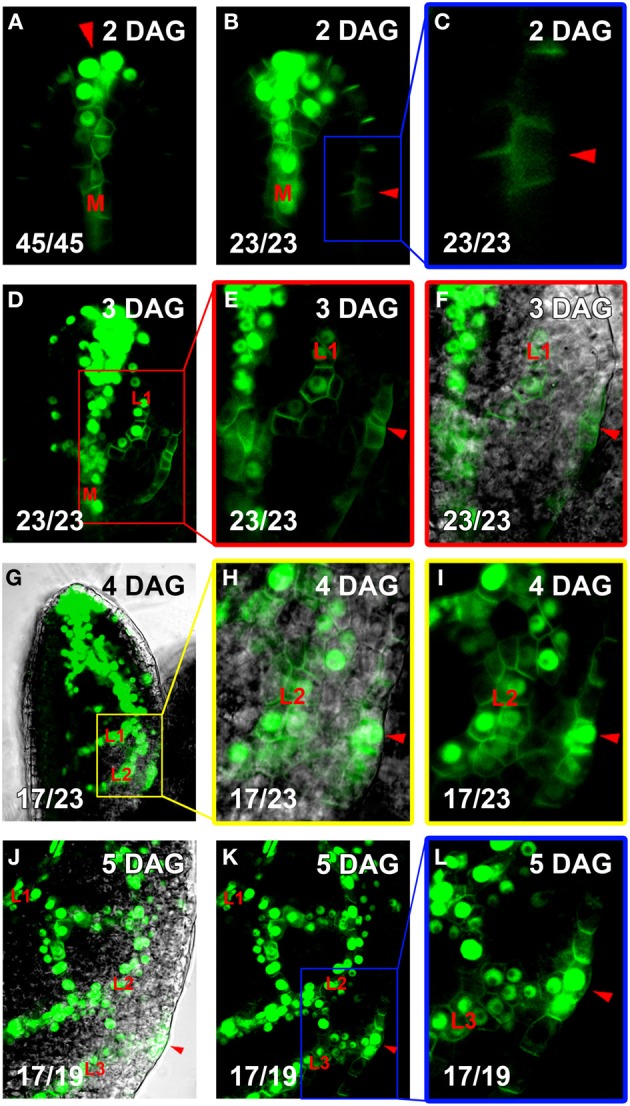
**Co-visualization of *DR5rev::VENUS:NLS* and *PIN1:GFP***. Top right, leaf age in DAG, bottom left, reproducibility quotient (Table 2). Scale bars: **(A–F**,**H–L)** 10 μm; **(G)** 20 μm. **(A–C)** Expression of *DR5rev::VENUS:NLS* (nuclear) and *PIN1:GFP* (plasma membrane). Note strong *DR5rev* at apical convergence point (CP, red arrowhead in **A**) and midvein (labeled **M** in **A,B**), but faint *DR5rev* in lateral CP the first loop (red arrowhead in **B**, enlarged in **C**). **(D,E)** At 3DAG, extension of *DR5rev* to first loop PED (labeled L1 in **D–F**). Red-boxed area in D enlarged in **(E,F)**, with DIC channel overlay in **(F)**. Lateral CPs marked by red arrowheads in **(E,F)**. **(G–I)** At 4DAG, extension of *DR5rev* expression to second loop PEDs (labeled L1 and L2, respectively, in **G–I**). Yellow-boxed area in **(H,I)**, with DIC channel overlay in **(H)**. *DR5rev* expression now present in the lateral CP (red arrowheads in **H,I**). **(J–L)** At 5 DAG, *DR5rev* extension to third loop PEDs (labeled L1, L2, and L3, respectively, in **J–L**). The same leaf area shown in **(K)** with DIC channel overlay in **(J)**. Blue-boxed area enlarged in **(L)**. *DR5rev* expression lateral CP of third loop (red arrowheads in **J–L**).

Intriguingly, wider and early branching hPEDs were not evenly distributed throughout the leaf lamina, but were significantly more frequent in the intercostal areas enclosed by the second and third loop than in the first loop (Table 1). To test whether this could be the result of higher auxin levels in the respective areas, we visualized expression of the *DR5* auxin response marker together with *PIN1* across the leaf lamina. As shown in Figure 5, second and third loops are in areas of *DR5* expression at their associated epidermal auxin convergence points (CPs), while the first loop is not. While no *DR5* expression was ever detected in the first loop CP (Figures 5B–F), strong *DR5* auxin response maxima were found in 17 of 23 second loop CPs (Figures 5G–I), and in 17 out of 19 third loop CPs (Figures 5J–L). If one interprets the *DR5* intensity at the CP as a reflection of the overall auxin exposure of the respective lamina area, this could mean that hPEDs formed in areas of apparent elevated auxin levels tend to be initiated as wider and more branched hPEDs. In conclusion, all hPEDs display a persistent tendency toward narrowing and their initial width is correlated to the apparent auxin levels in the respective leaf area. It seems very likely that hPEDs in all areas have similar ontogenies and that the observed width differences simply reflect different levels of *PIN1* expression in those areas (see Discussion).

**Table 1:** **Frequency of initially wider or branched hPEDs in the first, second and third intercostal areas (IAs) of the first leaf**.

	**#Wider hPEDs^a^**	**#Branched hPED^b^**	**Total # hPEDs observed**	**Frequency wider hPEDs**	**Frequency branched hPED**
1st IA	17	3	86	19.8%^**^	3.5%^*^
2nd IA	56	22	91	61.5%^**^	24.2%^*^
3rd IA	23	4	32	71.9%^**^	12.5%^*^
Total	96	29	209	45.9%^**^	13.5%^*^

a *Initially wider hPEDs were defined as hPEDs that were more than two cells wide at first appearance*.

b *Branched hPEDs were defined as freely-ending hPEDs that formed side branches*.

** *The distribution of wider hPEDs across the 1, 2, 3rd IA differs significantly from null hypothesis of even distribution to P = 0.001*.

* *The distribution of branched hPEDs across the 1, 2, 3rd IA differs significantly from null hypothesis of even distribution to P = 0.01. Significance was calculated using the Chi-squared test for a 2 × 3 contingency table, assuming two degrees of freedom*.

**Table 2:** **Features used as criteria in the calculation of reproducibility quotient**.

**Figure 6:**
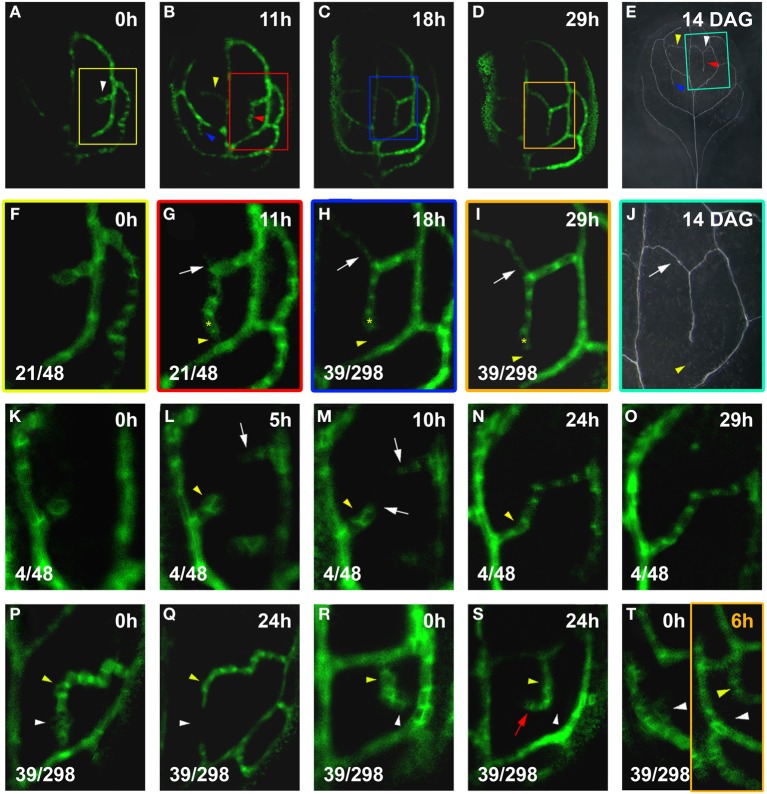
**hPED fusion and instability**. Top right time after first visalization (*t* = 0; **A–D**,**F–I**,**K–T**) or leaf age in DAG **(E,J)**. Bottom left, reproducibility quotient (Table 2). Scale bars: **(A–D)** 20 μm; **(E)** 1 mm; **(F–I**,**K–T)** 10 μm; **(J)** 0.5 mm. **(A–E)** *PIN1:GFP* expression and final vein pattern of the same leaf. Note the presence of one hPED at 0 h, and three new hPEDs at 11 h (arrowheads in **A,B**). Corresponding veins marked by arrowheads in **(E)**. **(F–J)** Enlargements of boxed areas in **(A–E)**, respectively. A freely-ending hPED **(F)**, has extended and fused with the first loop below (yellow arrowhead, **G**). Emerging side branch (white arrow in **G**), turned into a second connected hPED (white arrow in **H,I**). Lost *PIN1* expression (yellow arrowhead in **H,I**) resulting in the formation of one connected vein (white arrow in **J**) with a freely-ending, side branching vein (yellow arrowhead in **J**) in final vein pattern. Yellow asterisk marks same cell in **G–I**, as positional reference. **(K–O)** Freely-ending hPED branching from the first loop **(K)**, followed by second freely-ending hPED from the midvein (white arrow in **L**). White arrows in **(M)** mark extensions to both hPEDs. Further extension leads to fusion of hPEDs in **(N,O)**. Transient hPED widening (yellow arrowhead, **L,M**) followed by subsiding *PIN1* expression and narrowing (yellow arrowhead in **N**). **(P–Q)** Relatively higher *PIN1* expression in slightly elongated cells (yellow arrowhead) contrasted by polygonal cells with lower *PIN1* expression (white arrowhead). By 24 h **(Q)**, some of the polygonal cells have lost *PIN1* expression (white arrowhead), while the slightly elongated cells have maintained *PIN1* expression (yellow arrowhead), resulting in the formation of two freely-ending hPEDs. **(R–S)** Similar slightly elongated cells with high *PIN1* expression (yellow arrowhead) and polygonal cells with low *PIN1* expression (white arrowhead). By 24 h **(S)**, similar cell fate partitioning between polygonal (white arrowhead) and elongated cells (yellow arrowhead), resulting in the redirection of the freely-end of the hPED. **(T)** Ephemeral hPED with low *PIN1* expression in polygonal cells (**T**, white arrowhead left panel), replaced at 6 h (**T**, right panel) by new freely-ending hPED (yellow arrowhead).

**Figure 7:**
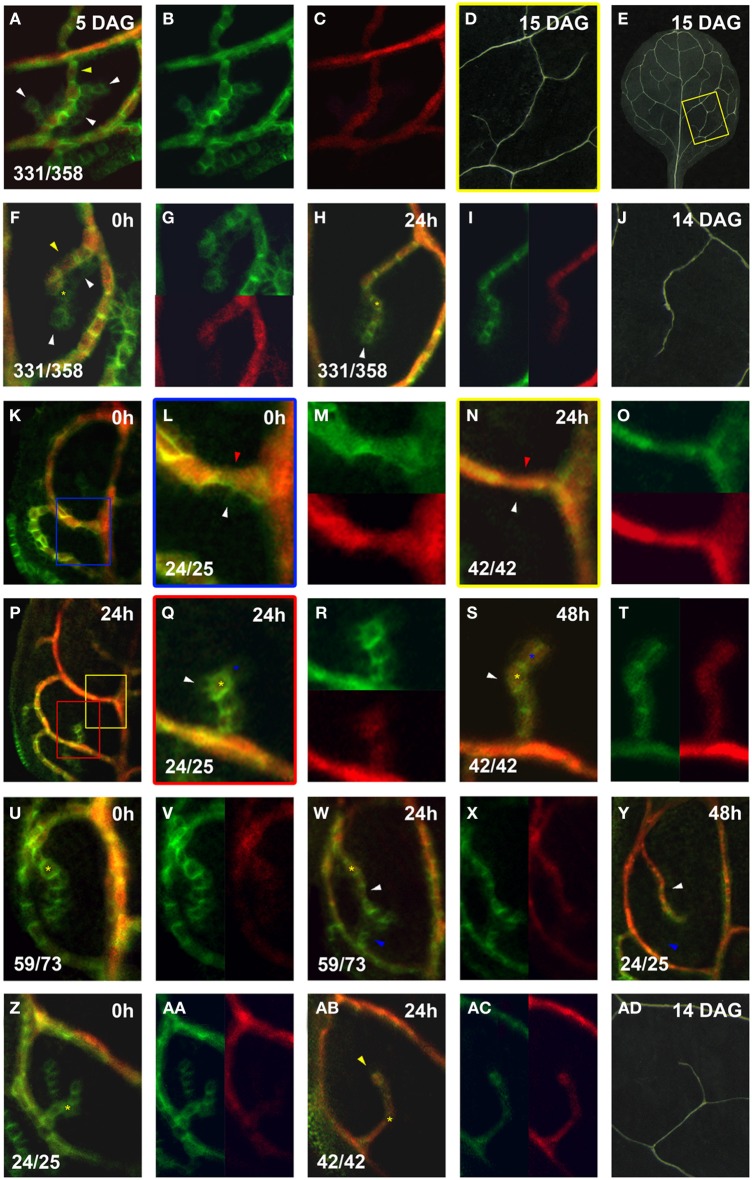
**Co-expression of *PIN1:GFP* and *Athb8::YFPer* during vein formation**. *PIN1:GFP* (green) and *Athb8::YFPer* (red). Top left, time after first imaging (*t* = 0; **F,H,K,L,N,P,Q,S,U,W,Y,AC,AD**) or at leaf age in DAG **(A,D,E,J,AD)**. Bottom left, reproducibility quotient (Table 2). Scale bars: **(A,F–H,L–N,Q–AB)** 10 μm; **(D,E)** 1 mm; **(J,AD)** 0.5 mm; **(K,P)** 20 μm. **(A–E)** Area enclosed by the third loop. Co- **(A)** and separate **(B,C)** expression of *PIN1:GFP* and *Athb8::YFPer*. Note *Athb8* confinement to central, high *PIN1* expressing, elongated cells (yellow arrowhead in **A**) and absence in lower *PIN1* expressing cells (white arrowheads in **A**). This pattern is reflected in the final vein pattern **(E)**, enlarged in **(D)**. **(F–J)** Area enclosed by the first loop.Co- **(F)** and separate (**G**, upper, lower) expression of *PIN1:GFP* and *Athb8::YFPer*. Initial **(F)**, *Athb8* expression proximal, nearest to attachment to lower order PED in elongated cells with higher *PIN1* expression (yellow arrowhead in **F**), and absent from polygonal cells with low *PIN1* expression at the edges and free ends of hPEDs (white arrowheads in **H**). Co- **(H)** and separate (**I**, left, right) expression of *PIN1:GFP* and *Athb8::YFPer*. Note that *Athb8* is now expressed in some terminal cells, as these have upregulated *PIN1* and elongated slightly (yellow asterisk in **H**; same cell labeled with yellow asterisk in **F**), but not yet in the newly added cell at the free end (white arrowhead in **H**). Vein corresponding to hPED in **F–I** in mature leaf **(J)**. **(K–M)** Leaf at 4 DAG (0 h; **K**). Blue-boxed area in **(K)** enlarged in **(L)**, and separated for *PIN1:GFP* and Athb8::YFPer expression in **(M)** (top, bottom). Expression of *Athb8* in elongated, *PIN1* expressing upper cells in first loop PED (red arrowhead in **L**), but absence from lower cells of the first loop PED that are polygonal with diminished *PIN1* expression (white arrowhead in **L**). **(N–P)** 24 h later visualization of leaf in **(K)**. Yellow-boxed area in **(P)** enlarged in **(N)** and split in separate expression channels in the top and bottom panels of **(O)**. Note persistence of *Athb8* expression in upper cells of the first loop (red arrowhead in **N**), while the lower cells of the first loop PED no longer express *PIN1* (white arrowhead in **N**). **(P–T)** Red-boxed area in **(P)** enlarged in **(Q)** and expression separated in the top and bottom panels of **(R)**. The central cells of this a newly emerging hPED with highest *PIN1* expression express *Athb8* (yellow asterisk in **Q**), while edge cells do not (white arrowhead in **Q**). **(S,T)** Same hPED at 48 h **(S)** and expression separated in left and right panels of **(T)**. Central *Athb8* expressing cells (yellow asterisk in **Q**) have persisted (yellow asterisk in **S**), while the majority of the edge cells without *Athb8* expression (white arrowhead in **Q**) have also lost *PIN1* expression (white arrowhead in **S**). By contrast, a cell at the free end of the hPED, which maintained *PIN1* expression and elongated now expresses also *Athb8* (blue asterisks in **Q,S**). **(U–Y)** Leaf at 4 DAG (0 h) with hPED extending from the first loop **(U)**. Expression channels separated in the left and right panels of **(V)**. Expression of *Athb8* in central, proximal cells (yellow asterisk in **U**). **(W)** Same leaf as in **(U)** and expression separated in the left and right panels of **(X)**. More distal cells have elongated, upregulated *PIN1* and express *Athb8* (white arrowhead in **W**). [Same cell marked by yellow asterisk in (**U,W**) as positional reference). A new freely-ending hPED (blue arrowhead in **W**); the cells of this new hPED does not express *Athb8*. **(Y)** Same hPED as in **(W)** at 48 h. Note that the upper hPED expressing *Athb8* has persisted (white arrowhead in **Y**), while the lower without Athb8 expression (blue arrowhead in **W**) has also lost *PIN1* expression at 48 h (blue arrowhead in **Y**). **(Z–AD)** Leaf at 5 DAG (0 h) with branched, freely-ending hPED from the first loop **(Z)**. Expression displayed separately in the left and right panels of **(AA)**. No *Athb8* expression in the hPED at 0 h **(Z,AA)**. **(AB)** Same sample at 24 h and separately in **(AC)** (left/right). Cells of upper branch (yellow arrowhead) elongated and express *Athb8*, those of lower branch have also lost *PIN1* expression. A corresponding vein appears in the mature leaf **(AD)**.

Figure 2
G–K: 82/101 hPEDs underwent the transition from preprocambium to procambium (as defined in text) during the imaging interval
Figure 3
A–J: Out of 44 extending hPEDs, in 35 cases extension was due to terminal addition
Figure 4
A,B: 29/209 PEDs formed side branches while still freely ending
C–E: 92/209 hPEDs observed were initially >2 cells wide and narrowed to domains 1–2 cells wide during the imaging interval
K–O: 4/48 connected hPEDs observed were formed via the fusion of two freely-ending hPEDs
Figure 5
A: Strong *DR5rev::VENUS* expression was present in 45 out of 45 apical CPs observed
B–F: *DR5rev::VENUS* expression was absent from 23 out of 23 first loop-associated lateral CPs
G,H: *DR5rev::VENUS* expression was present in 17 out of 23 second loop-associated lateral CPs
J–K: *DR5rev::VENUS* expression was present in 17 out of 19 second loop-associated lateral CPs
Figure 6
F–G: 21/48 connected hPEDs observed were formed via the fusion of a freely-ending hPED with a static PED.
H–I: 39/298 hPEDs observed exhibited loss of *PIN1* expression in domains of 3 cells or more
K–O: 4/48 connected hPEDs observed were formed via the fusion of two freely-ending hPEDs
P–T: 39/298 hPEDs observed exhibited loss of *PIN1* expression in domains of 3 cells or more
Figure 7
A,F: In 331/358 hPEDs, axialized cells with high *PIN1* expression expressed *Athb8::YFPer*, while polygonal cells with low *PIN1* expression did not
L,Q,Y: In 24/25 hPEDs, *Athb8* was not expressed in edge cells with centrobasal PIN1 polarity that disappear
N,S,Z: In 42/42 hPEDs, *Athb8* was expressed in central cells with basal PIN1 polarity that persist
U,W: Out of 73 hPEDs that showed differences in *Athb8::YFPer* expression along the strand, 59 hPEDs exhibited stronger *Athb8* expression in cells at the base of the hPED vs. cells at the free end

### Formation of higher-order vein networks via fusion of freely ending hPEDs

The formation of closed vein networks via the fusion of incipient freely-ending vein segments has previously been observed in the lower order vein loops (Scarpella et al., 2004, 2006) and with regard to other vascular cell state markers in higher-order veins (Sawchuk et al., 2007). In order to test whether connected veins are generally formed via the fusion of antagonistically oriented auxin-transport routes, we visualized the ontogeny of connected hPEDs. Using imaging intervals of less than 24 h, we followed the development of 25 connected hPEDs (Figures 6F,G,K–O). All of these were formed in one of two ways: (1) Through the fusion of the extending hPED with a pre-existing lower order PED, e.g., hPED fuses with a loop PED (21/25 or 84% of cases; Figures 6F,G); (2) Through fusion of two extending, freely-ending hPEDs (4/25 or 16% of cases; Figures 6K–O). Other possible modes of hPED fusion—such as the formation of an island of *PIN1* expression that extends outwards and eventually connects to another hPED at each end—were never observed. Therefore, we conclude that connected hPED are usually formed via the fusion of an extending, freely ending hPED with a static, pre-existing PED or another extending hPED. Furthermore, newly formed connected hPEDs were on average only 7.1 cells long (*n* = 31), indicating that hPEDs become connected in an environment of relatively small cell numbers (see Discussion).

The gentle confocal settings required for live imaging (see Methods) and leaf thickness at the time of connected hPED formation made it difficult to clearly determine PIN1 polarity in all cells of newly connected hPEDs. However, in all 9 cases in which hPED fusion had occurred relatively early in leaf development and PIN1 polarity could be distinguished in the cells of the hPED pre-and post-fusion, it was observed that before fusion, all cells of the freely-ending hPED(s) had PIN1 polarity directed toward their connecting lower order vein (Figures 6K–M). Post-fusion, these same cells retained their PIN1 polarities, while one of the most recently added cells was bipolar (Figures 6N,O). The remaining newly added cells had PIN1 polarity directed toward the nearest connecting lower order vein. These data indicate that hPED fusion does not involve reversals in the PIN1 polarities. Rather, they suggest that a single bipolar cell is formed concomitant with and at the site of fusion of the two inversely polarized freely-ending hPEDs.

### hPED stability

Live imaging visualizes the actual *PIN1* expression dynamics of all subepidermal cells during vascular patterning, regardless of whether these cells are ultimately recruited into the final vein pattern. Our analysis surprisingly but consistently revealed that hPEDs are often unstable soon after their formation: *PIN1* expression in cells of the hPED can be transient, resulting in connections that are made and then lost (Figures 6G vs. 6H, 6P vs. 6Q), and hPED sections that extend and then disappear (Figures 6R vs. 6S, 6T left panel vs. 6T right panel). We observed loss of *PIN1* expression in domains of three cells or more in 39/298 hPEDs (13%) visualized at intervals of 24 h or less over a period of 3 days. These dynamic changes in *PIN1* expression were only observed in hPED cells that were polygonal in shape and had relatively low *PIN1* expression (22/22, Figures 6G vs. 6H, 6P vs. 6Q, 6R vs. 6S). Cells with high *PIN:GFP* expression or elongated cell shape consistently maintained stable *PIN1* expression during live imaging (63/63, Figures 6G–J).

The downregulation of *PIN1* expression in certain cells of a hPED did not seem to be due to a breakdown of the vein patterning mechanism in the respective leaf area, since new hPEDs were regularly formed in the close vicinity of where a hPED section had just disappeared (Figures 6R,S,T left panel vs. T right panel). These observations suggest that during normal higher order vein patterning, auxin transport routes are initially dynamically changing, and only routes carrying sustained auxin flux over time differentiate as vascular strands.

In order to better understand the regulation of hPED dynamics, we asked whether increased hPED stability coincided with the onset of expression of one of the earliest markers of vascular identity, the preprocambial marker *Athb8*. We observed that *Athb8::YFPer* was expressed within the central cells of narrowing PEDs, such that the domain of *Athb8::YFPer* expression never exceeded the bounds of the *PIN1* expression domain (Figures 7A–C,F,G,Q–R). The onset of *Athb8::YFPer* expression within cells generally slightly preceded the cells' transition from polygonal in shape with low *PIN1* expression to a more axialized, rectangular shape and increased *PIN1* expression: Most PEDs composed of axialized cells with high *PIN1* expression expressed *Athb8::YFPer* (197/211), and conversely, most PEDs composed of polygonal cells with low *PIN1* expression did not express *Athb8::YFPer* (134/147; Figures 7A–C,F–I,Q–T). This correlation was observed in PEDs of all orders (midvein, second order loops and higher-order PEDs; Figures 7K,P). However, both axialized PED cells lacking *Athb8::YFPer* expression (14/211) and polygonal PED cells expressing *Athb8::YFPer* (13/147) were also observed at low frequency, indicating that *Athb8::YFPer* expression does not perfectly correlate with this cell shape transition. Nonetheless, the timing of onset and spatial pattern of *Athb8::YFPer* expression made it the best available marker of cells that have undergone vascular specification.

Within narrowing loop and higher order PEDs, *Athb8::YFPer* was always strongly expressed in the central cells with basal PIN1 polarity that persisted over time (observed in 21/21 cases), and was almost always absent from the edge cells with central PIN1 polarity that disappear (observed in 21/22 cases, Figures 7L–O,Q–T). In agreement with this, the majority of hPEDs that expressed *Athb8:YFPer* persisted over 24–48 h of live imaging (58/60). Conversely, most hPEDs that disappeared during the same live imaging period did not express *Athb8::YFPer* (20/22). In two cases, however, *Athb8::YFPer* appeared to be expressed in an polygonal cell that later disappeared from the hPED (data not shown), indicating that *Athb8::YFPer* could not be used as a marker of irreversible vascular commitment. We nonetheless enquired whether expression of *Athb8::YFPer* in an incipient vascular strand was correlated with enhanced stability of *PIN1* expression and persistence of the strand onto final vascular differentiation. Out of 292 hPEDs examined, we observed that 26 hPEDs (8.9%) did not differentiate as xylem strands in the final vascular pattern of the leaf. Out of these 26 disappearing hPEDs, 21 (80.8%) were hPEDs that expressed *PIN1* alone without *Athb8::YFPer*, whereas 5 (19.2%) expressed both *PIN1* and *Athb8:YFPer*, indicating that hPEDs expressing *Athb8* are significantly more likely to differentiate as mature veins (*P* = 0.0017; Figures 7U–Z,AA–AD). A likely possibility is that the five *Athb8* positive hPEDs that failed to differentiate as xylem strands were specified as procambium and maintained *Athb8* expression but did not complete vascular differentiation. However, lack of a suitable procambial marker made the detection of these cells at this late stage of leaf development very difficult. Taken together, these data suggest that vascular specification occurs progressively in PEDs, such that cells in the center of narrowing PEDs that are exposed to the highest auxin over time upregulate factors that stabilize their *PIN1* expression and promote their differentiation into vascular stem cells.

## Discussion

### Reiterating auxin-flow dynamics in higher-order vein formation

The selection process carving out the pattern of procambial strands from the initially uniform populations of subepidermal cells in leaf primordia is ideally suited for mathematical as well as experimental investigation of self-regulatory patterning processes in plants. Moreover, a number of auxin-associated gene expression markers allow for the recording of early temporal-spatial events at incipient stages of vein formation, which serve as powerful visualization tools guiding mathematical modeling of the process (Scarpella et al., 2006; Sawchuk et al., 2007). Along these lines, it has previously been shown that auxin convergence points in the epidermis, visualized through *PIN1:GFP* expression, are associated with the positioning of the midvein and the second order vein loops, which together set-up the scaffold on which the higher vein network is elaborated (Reinhardt et al., 2003; Scarpella et al., 2006). For those few vein classes that form reproducible patterns, and whose ontogeny can thus be reconstructed from fixed samples, it was further concluded that at the incipient stage of mere *PIN1* expression, connected veins are invariably derived from freely-ending veins, each of which with unidirectional *PIN1* subcellular polarity. Further, it was observed that the formation of a single bipolar cell was firmly associated with each connected PED, suggesting that it marks the position of fusion of two, originally separate PEDs of opposite polarity (Scarpella et al., 2006). In this study, we have addressed the generality of the preceding PIN1 expression dynamics using a live-imaging protocol and found very similar features preceding the formation of connected veins in all vein classes. Our findings not only support common principles across all vein classes, they also reveal a highly ephemeral nature of early PEDs followed by the selection of connected vein paths within fields of relatively small numbers of ground meristem cells, which can be expected to be important for the probability at which PED fusions can occur (Scarpella et al., 2006). Although there does not seem to be a uniform auxin distribution throughout the leaf lamina at any stage of development, our results leave open whether auxin gradients or “with the flux” mechanisms (Bayer et al., 2009) or both may have causative roles.

**Figure 8:**
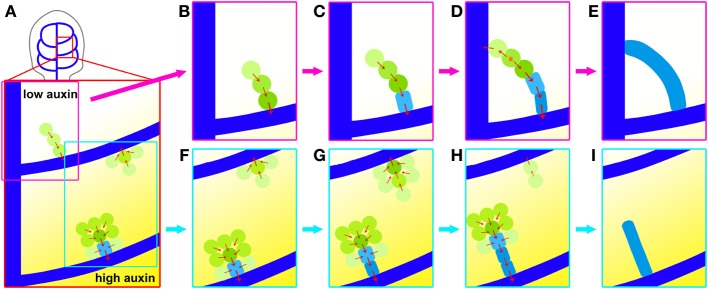
**Dynamics of higher-order vein patterning. (A)** top: location of area enlarged below, hPED forming in higher auxin environment (bottom examples in stronger yellow shading) more likely to be wider and have strong *PIN1* (green) and some *Athb8* (light blue) expression. **(B,C)** hPED extension and fusion via terminal addition; from preexisting PEDs (dark blue) primarily via the upregulation of *PIN1* in new cells at the free end. **(C–E)**. Formation of connected hPED often by fusion at free end with another PED, generating bipolar cell (orange star). Onset of *PIN1* upregulation and cell elongation coincides with vascular commitment, indicated by *Athb8* expression (light blue). **(F–I)** hPED formation from domain >2-cells wide and hPED competition. Cells at edges display inward *PIN1* polarity followed by loss of *PIN1* expression, thereby narrowing the hPED **(F,G)**. Concomitantly, central cells upregulate *PIN1*, elongate and express *Athb8* (light blue), indicating their vascular selection. Local competition between hPED for auxin may contribute to observed dynamic instability **(G–I)**. A faint hPED is more likely to disappear when emerging near an advanced wide hPED with committed vascular cells in the center.

Visualizing the actual sequence of earliest stages in the formation of individual leaf veins revealed that higher order vein network is laid down through reiterating phases of hPED initiation, followed by hPED narrowing and expression of permanent vascular markers from Ath8 expression to cell shape changes. During the first phase, hPED formation, new hPED are initiated as freely ending domains that extend from the pre-existing PEDs via terminal addition (Figures 8B,C,F,G). In a minority of cases, the extending freely-ending hPED comes in close proximity to a static lower order PED or, more rarely, with other extending hPEDs, and a fusion occurs between these two PEDs (Figures 8C,D). Live imaging of fusing hPEDs at short time intervals indicates that prior to fusion, the cells of the hPED are uniformly polarized toward the connecting lower order vein (Figures 8B,C), while immediately post-fusion, the hPED is composed of two sections of opposite polarity linked by a newly added bipolar cell (Figure 8D). These recurrent observations indicate that bipolar cell formation is a late event during connected vein formation that occurs in a single cell located between two hPEDs with PIN1 polarities directed away from this cell, possibly toward two neighbouring divergent auxin sinks. These bipolar cells remain stable in the growing primordium and the co-visualization of PIN polarity and cytoskeletal orientations may be necessary to better understand the formation and maintenance of cells with dual polarity. Recent results (Heisler et al., 2010) make it unlikely that the stability of PIN polarity in bipolar cells could be a directly enforced by the microtubular cytoskeleton. Instead, one has to assume a lasting orienting mechanism acting from inside or outside the cell to stabilize PIN polarity.

During the strand formation phase of higher order vein patterning, hPEDs may either initially appear as wide or narrow, branched or unbranched, depending on their local auxin environment. In areas of the first leaf with higher predicted auxin levels, as judged from the *DR5* expression levels at the corresponding CPs, such as the areas enclosed by the *DR5* maximum-associated second or third loops, a greater proportion of freely-ending hPEDs are branched or more than two cells wide, as compared to the area enclosed by the first loop, which is not associated with a *DR5* auxin response maximum (Figure 8A). Since *PIN1* expression increases with increasing auxin levels, it could be that the higher auxin levels in the second and third intercostal areas result in a greater fraction of cells that express *PIN1:GFP* at levels above the minimum detection threshold of our visualization system. Thus, in areas with high apparent auxin levels, we were able to visualize *PIN1* dynamics in a greater number of cells participating in the higher order vein patterning process.

While *PIN1* expression was sometimes detected in broad domains during hPED formation, these hPEDs invariably resolved into narrow strands of vascular precursors during the second phase of higher order vein patterning: hPED narrowing and definition of the vascular prepattern (Figures 8F–I). Live imaging allowed us to study this phase in detail. First, the cells within wide hPEDs were partitioned into two fates: Cells at the edges of the domain relocalized PIN1 toward the cells at the domain center (Figures 8F–H). These edge cells eventually downregulate *PIN1* and disappear from the domain, resulting in a hPED that is uniformly narrow. The cells in the narrow, central domain, on the other hand, upregulate *PIN1* and elongate and/or divide parallel to the axis of the strand, thereby acquiring high auxin transport capacity and an axis of cell elongation parallel to the predicted axis of auxin flow, two traits of vascular precursors (Sachs, 1981, 1991). The degree of expression of these two traits initially correlates with the cell's position within the freely ending strand: In most strands, there is a smooth gradient in cell length and *PIN1* expression such that cells at the base of the strand that are predicted to receive the most auxin and have been exposed to high auxin flux the longest are typically most elongated and express the most *PIN1* while cells at the free end are shortest and express the least. Eventually, these same cells express *Athb8::YFPer* indicating their selection as vascular precursors (Figures 8F–H). Auxin is known to induce cell elongation (Sachs, 1981) and to regulate the expression of *PIN1* (Vieten et al., 2005), and *Athb8* (Baima et al., 1995; Mattsson et al., 2003; Donner et al., 2009). Taken together, these data suggest that auxin directly promotes the acquisition of elongated cell shape, high auxin transport capacity and vascular gene expression by vascular precursors.

### hPED instability reveals the dynamic nature of leaf vascular patterning

Competition between neighbouring cells for auxin flow, leading ultimately to the upregulation of *PIN1* in a narrow file of cells and its downregulation all others is apparent during the narrowing of PEDs of all orders, particularly under conditions of experimentally elevated auxin (Scarpella et al., 2006). In contrast, competition for auxin flow between spatially distant cells is not readily apparent during undisturbed lower order vein patterning, and there was previously no evidence to suggest that such competition could dramatically alter where a vein would form.

Live imaging unexpectedly revealed that in 13% of hPEDs, short domains disappeared soon after their formation, leading to dramatic changes in the connectivity and architecture of the vascular pre-pattern (Figures 8F–I). The disappearance of a part or a whole hPED was regularly accompanied by the formation of a new branch or nearby hPED, suggesting that early hPED instability was not simply caused by the stress of live imaging, but was rather a part of normal vein patterning. *PIN1* expression was unstable in early hPED cells that lacked signs of vascular cell selection (i.e., with low *PIN1* expression and polygonal shape), that usually did not express *Athb8::YFPer* (Figures 8G–I). In contrast, hPEDs expressing *Athb8::YFPer* showed enhanced stability and greater likelihood to differentiate into veins (Figures 8G–I). These observations are in agreement with recent reports that *Athb8* is required to stabilize preprocambial cell specification against perturbations in auxin transport (Donner et al., 2009). Thus, hPED instability suggests that competition for auxin flow between different groups of cells may determine which of these forms a stable auxin transport route, and ultimately a vein. Prolonged auxin transport within a hPED appears to trigger *Athb8:YFPer* expression, stabilizing *PIN1* expression within the hPED against subsequent dynamic, auxin fluctuations in its environment (Figures 8F–I, Donner et al., 2009).

Initial hPED instability may allow the plant to select the optimal vascular pattern for each leaf. Leaf sizes across genetically identical plants can vary dramatically depending on environmental factors, such as water, light and nutrient availability (Jurik et al., 1982). The repeated deployment of a rigidly genetically specified higher order vein pattern in all leaves would be highly maladaptive, since larger leaves require more higher order veins to adequately meet their nutrient transport requirements, while smaller leaves need fewer. Thus, the fittest flexible vein patterning mechanism would be predicted to be capable of sensitively matching the reticulation of the vascular network to the final size of the local leaf areas. Such precise matching could be achieved simply if proliferating cells in the leaf primordia produce a vein promoting signal, such as auxin: Leaf areas with sustained, high cell proliferation, which will ultimately require the greatest vascular transport capacity, will likewise have the greatest potential to generate stable auxin transport routes and vascular strands, out-competing leaf areas with relatively lower rates of cell proliferation. Early hPED instability may thus allow the leaf to integrate potentially fluctuating cell proliferation signals from many cells in a given area before selecting the most appropriate vascular pattern for that area.

### Toward a universal auxin transport-dependent vein patterning mechanism

The evolution of vein modeling is driven by advances in mathematical modeling, visualization technology and entries from genetic/experimental manipulation (Berleth et al., 2007). The highly reproducible regularities observed in this study, as formalized in Figure 8, are consistent with a signal flow mechanism and the narrowing of *PIN1* expression domains, is strongly suggestive of canalized auxin-transport as proposed in pioneering studies also as a means for the coordinated development of the lamina and a corresponding, functioanl venation pattern (Sachs, 1981, 1989). The mathematical and cell biological description of the process will be subject to constant re-interpretation, and new visualization tools may eventually reveal the participation of virtually all subepidermal cells at the very earliest stages of selection. Nevertheless, it is worthwhile to conceptualize observed regularities in the light of currently available cell biological information, which suggest the following interpretation:

During the hPED formation phase of higher-order veins, auxin signals induce the formation of hPEDs as initially freely-ending domains that branch from the previously formed midvein and loops, and direct auxin flow into these established sinks (Figure 8). The number of cells recruited into a hPED depends on local auxin levels (Figure 8A). Freely ending hPEDs extend through the upregulation of *PIN1* in new cells at the free end. When an extending hPED comes into close proximity with another hPED, such that a small group of cells is exposed to two strong, divergent auxin flows at close proximity to one another, a connected hPED is formed with a single bipolar cell (Figures 8C,D).

During the hPED narrowing and definition phase of higher order vein patterning, hPEDs are restricted to narrow domains that show the gradual acquisition of vascular traits. In wide hPEDs, edge cells orient PIN1 polarity toward neighbouring cells with highest auxin response. This results in the drainage of auxin from the edge cells into the central cells. Auxin depletion from edge cells negatively regulates their *PIN1* expression levels, resulting in loss of *PIN1* expression in these cells and the narrowing of the hPED (Figures 8F–H). In contrast, the central cells experience elevated auxin signaling. High auxin signaling promotes the vascular specification of the central cells by increasing *PIN1* expression, thus maintaining this positive feedback cycle, and inducing cell elongation and the expression of vascular genes, such as *Athb8* (Figures 8F–I). Expression of *Athb8* in turn stabilizes *PIN1* expression in the central cells of the hPED against future perturbations in auxin transport, and coordinates the transition to procambial identity (Figures 3.8 G–I, Donner et al., 2009). If a hPED is outcompeted or deprived of auxin transport before the onset of vascular specification, it may lose *PIN1* expression and adopt a non-vascular fate (Figures 8G–I, Scarpella et al., 2004). Thus, only hPED that carry sustained, elevated auxin flux acquire vascular identity and differentiate as veins.

This interpretation underscores the basic identity of the vein patterning mechanism that generates the midvein, lower-loop domains and the higher-order veins: These vein types are initiated as freely-ending domains in continuity with pre-existing PEDs. Both elongate via terminal addition and fuse with the formation of bipolar cells. In areas of elevated auxin, both appear as initially wider PEDs that exhibit the same the intrinsic narrowing capacity (Figure 8). These basic similarities suggest that the differences observed between lower order and higher order vein patterning (e.g., the more regular pattern of midvein and loops vs. extremely variable pattern of higher-order veins; the initial instability of hPEDs vs. the apparent stability of midvein and loop PEDs) are not due to differences in the patterning mechanism that generates these veins but more likely the different signal environments in which this mechanism operates. The midvein and loop PEDs are formed in areas of relatively few cells, in association with strong, localized auxin sources (namely, the auxin convergence points in the marginal epidermis) and/or sinks (such as the midvein). The strength and localized nature of these sources and sinks and the limited number of cells available to respond to them may account for the observed stability and regularity of lower order vein patterning. In contrast, higher order PEDs form in areas composed of many cells, where the auxin sinks provided by the lower order veins are numerous and spatially distributed, and the auxin sources (putatively derived from the subepidermal cells) may be more diffuse and fluctuating. One would predict that PEDs formed in this environment would be more variable in term of positioning, architecture (straight or branched) and stability than the lower order veins, even if the rules that regulate *PIN1* upregulation, downregulation and localization are the same for both PED types.

Our data demonstrate there is not only a reproducible ontogeny from wide to narrow hPEDs, but also one between high *PIN1* and expression in those central domains and expression of *Athb8::YFPer*, which marked vascular commitment with near 100% reliability (266/271, 98.15%). Because variability among transgenic reporter genes can easily account for small differences, these data are compatible with a stringent link between expression of the *Athb8::GUS* marker and vascular commitment (Scarpella et al., 2004) and both markers are clearly highly suitable for all practical purposes. The emerging picture suggested by our data comprises a highly dynamic and often ephemeral marker, *PIN1*, whose eventual elevated and sustained expression then leads to the expression of the vascular commitment marker, *Athb8*, generating a completely unpredisposed, yet continuous network pattern. These features characterize higher-order venation patterning as a highly informative self-regulatory process, in which important molecular determinants can be recorded at high spatial and temporal resolution and whose potential in the breadth of plant species remains to be explored.

### Conflict of interest statement

The authors declare that the research was conducted in the absence of any commercial or financial relationships that could be construed as a potential conflict of interest.
